# T-cell receptor variable region usage in Chagas disease: A systematic review of experimental and human studies

**DOI:** 10.1371/journal.pntd.0010546

**Published:** 2022-09-15

**Authors:** Thaiany Goulart de Souza-Silva, Kenneth J. Gollob, Walderez O. Dutra

**Affiliations:** 1 Institute of Biological Sciences, Department of Morphology, Federal University of Minas Gerais, Belo Horizonte, Minas Gerais, Brazil; 2 Hospital Israelita Albert Einstein, São Paulo, Brazil; 3 Instituto Nacional de Ciência e Tecnologia em Doenças Tropicais, Belo Horizonte, Minas Gerais, Brazil; University of Texas at El Paso, UNITED STATES

## Abstract

T cells recognize their ligand, the peptide major histocompatibility complex (MHC), via the T-cell receptor (TCR), which is composed of covalently linked α and β or γ and δ chains. This recognition is critical for T-cell ontogeny and controls the selection, activation, and function of T lymphocytes. Specific TCR αβ variable regions have been associated with immunopathogenesis of Chagas disease. Here, we present a systematic review that compiles experimental in vivo and human data regarding the preferential expression of variable alpha (Vα) and variable beta (Vβ) chain regions in *Trypanosoma cruzi* infection. The original studies indexed in PubMed/Medline, Scopus, and Web of Science databases were screened according to the PRISMA strategy. The analysis showed that expression of TCR Vα subfamilies were evaluated in one human study, and, unlike TCR Vβ, TCR Vα presented a more restricted usage. Despite the great variability in the usage of TCR Vβ regions in human Chagas disease, a down-regulation of TCR Vβ5 expression by T cells from patients in the acute phase of the disease was shown. Opposingly, this TCR region was found overly expressed in CD4+ T cells from chronic Chagas patients. It was also demonstrated that murine Vβ9+ T cells derived from nonlymphoid organs of *T*. *cruzi*-infected animals had a modulatory profile, while splenic Vβ9+ T cells produced inflammatory cytokines, indicating that although they display the same TCR Vβ region usage, these cells are functionally distinct. Despite the limitations of few papers and year of publication of the studies, compiling the data derived from them reveals that further investigation of TCR usage will point to their potential role in protective or pathogenic responses, as biomarkers of disease progression, and in the search for dominant peptides potentially useful for the development of vaccines or therapies.

## Author summary

Chagas disease is a neglected tropical disease, caused by infection with *Trypanosoma cruzi*. Differential expression of certain T-cell receptor (TCR) variable regions has been associated with the immunopathogenesis of Chagas disease. Here, we present a systematic review that compiled experimental in vivo and human data regarding the preferential expression of TCR alpha and beta chain variable regions in Chagas disease. The original studies indexed in the PubMed/Medline, Scopus, and Web of Science databases were screened according to the PRISMA strategy. Despite the great variability in the use of TCR Vβ in *T*. *cruzi* infection, the outcomes indicate that there is a down-regulation of TCR Vβ5 expression in T cells from patients in the acute phase of Chagas disease. However, this region is preferentially expressed by CD4+ T cells from chronic Chagas patients. Additionally, it has been demonstrated that murine Vβ9+ T cells derived from nonlymphoid organs displayed a modulatory profile, while splenic Vβ9+ T cells produced inflammatory cytokines, indicating that although they express the same TCR Vβ region, these cells are functionally distinct. Information on TCR expression, specificity and function have critical impact on vaccine design.

## Introduction

The cell-mediated immune response is essential for host defense against invading pathogens and for maintaining immune homeostasis. However, when uncontrolled, it can generate immunopathology by exacerbating the initial damage triggered by the pathogen. After an infection, naïve T cells are activated and differentiate into heterogeneous and effector populations that directly or indirectly mediate pathogen clearance. The CD4^+^ T helper (Th) cells can differentiate into distinct subpopulations, such as Th1, Th2 [1], Th17 [2,3], Th9 [4], Th22 [5], and T regulatory (Treg) [6]. These subpopulations display distinct cytokine patterns and may perform antagonistic functions. In addition, under certain circumstances, CD4^+^ T cells can also exert effector functions such as cytotoxicity [7,8]. CD8^+^ T cells have a fast and robust proliferation rate [9] and are classically known for their cytotoxic role, thus being critical in eliminating intracellular pathogens through the expression of molecules with cytotoxic properties, such as granzymes and perforin [10]. In addition to conventional CD4^+^ and CD8^+^ T cells, a minor but functionally active T-cell subpopulation that do not express the CD4 or CD8 coreceptors, hence named double-negative T cells, exist [11]. A key characteristic shared by all T-cell subpopulations is the expression of a T-cell receptor (TCR) through which they typically recognize peptide, glycolipid antigens, or superantigens bound to major histocompatibility complex (MHC) or MHC-like molecules.

The TCR is a heterodimer composed of covalently bound α and β glycoprotein chains (95% of peripheral T cells) or γ and δ chains, which are involved in the recognition of the peptide MHC in most cases. Each TCR chain consists of a constant domain (C) anchored to the plasma membrane and a variable domain, containing 3 highly variable complementarity-determining regions (CDRs) [12,13]. The genes of the α and β chains are generated by the somatic recombination of the subregions V (variability) and J (junction), or V, D (diversity), and J, respectively, which provide the TCR with its fine specificity and also serve as an identifier of T cell ancestry [14,15]. TCR analysis provides information on antigen specificity, being a powerful tool to study the pathogenesis of human diseases including parasitic infections, such as malaria, leishmaniasis, and Chagas disease.

Chagas disease is a neglected tropical disease, caused by infection with the protozoan *Trypanosoma cruzi* and affects about 6 to 7 million people worldwide [16]. The transmission occurs mainly through the host’s contact with feces and urine of the infected triatomine vector, although oral transmission, blood transfusion, organ transplant, and congenital transmission are considerably important forms of infection [17–19]. In the acute phase of the disease, patients may manifest nonspecific clinical signs and symptoms [20] and have elevated parasitemia. Among the patients who reach the chronic phase, about 60% to 70% remain asymptomatic for years and are classified as belonging to the indeterminate clinical form. However, approximately 30% of chronically infected individuals develop the cardiac clinical form, which is characterized by the presence of a cardiomyopathy, resulting in an intense inflammatory reaction and causing high morbidity and mortality [17,21].

Both CD4^+^ T cells and CD8^+^ T cells play an important role in orchestrating the immune response and in disease progression [22,23]. It has been demonstrated that certain variable regions of the TCR seem to be associated with the immunopathogenesis of Chagas disease [24,25]. Our previous studies in human Chagas disease have shown that the frequency of Vβ5^+^ CD4^+^ T cells is decreased in acute infection and increased in the peripheral blood of chronic Chagas patients [24,25]. Thus, there is a distinct expression of certain TCR variable regions during Chagas disease, and this difference may be correlated with the stage of infection and clinical outcome.

Here, we present a systematic review that retrieved experimental (in vivo) and human data to understand which α- and β-chain variable region TCRs are preferentially expressed (or down-regulated) upon *T*. *cruzi* infection. Additionally, the methodological characteristics of the studies were assessed, indicating potential sources of risk of bias. This systematic review may guide new research toward functional and phenotypic analysis of cell populations expressing a specific TCR, which may emerge as a marker of disease severity. Moreover, TCR analysis can be used as a strategy to identify the antigens that these cells respond to, which may drive prophylactic or therapeutic strategies.

## Methods

This systematic review was constructed according to the Preferred Reporting Items for Systematic Reviews and Meta-Analyses (PRISMA) [26]. The methodologic strategy was registered at the International Prospective Register of Systematic Reviews–PROSPERO (registration number: CRD42020213115).

### Search strategy

The search strategy was set on 2 levels: (i) bibliographic search using the electronic databases MEDLINE (PubMed platform: https://www.ncbi.nlm.nih.gov/pubmed), Scopus (https://www.scopus.com/home.uri), and Web of Science (http://apps-webofknowledge.ez27.periodicos.capes.gov.br); and (ii) according to the reference list of relevant studies identified on primary search [27]. The filters were constructed following 2 criteria: Chagas disease AND TCR repertoire (S1 Table). Initially, the filters were built for PubMed platform, following the hierarchical distribution of the MESH terms (www.ncbi.nlm.nih.gov/mesh). The same search strategy used on PubMed platform was adapted and applied in the Scopus and Web of Science platforms. In order to ensure the retrieval of indexed studies and those that have been published or are under indexing process, the descriptors and keywords were combined with the Boolean operators AND/OR, just like the search algorithms [MeSH Terms] and [TIAB] [28,29]. All in vivo preclinical and human studies were analyzed for eligibility, without language restriction or publication date. The reference list of each eligible article was checked manually to find relevant publications to the guiding question of this systematic review.

### Selection of studies

**Fig 1:**
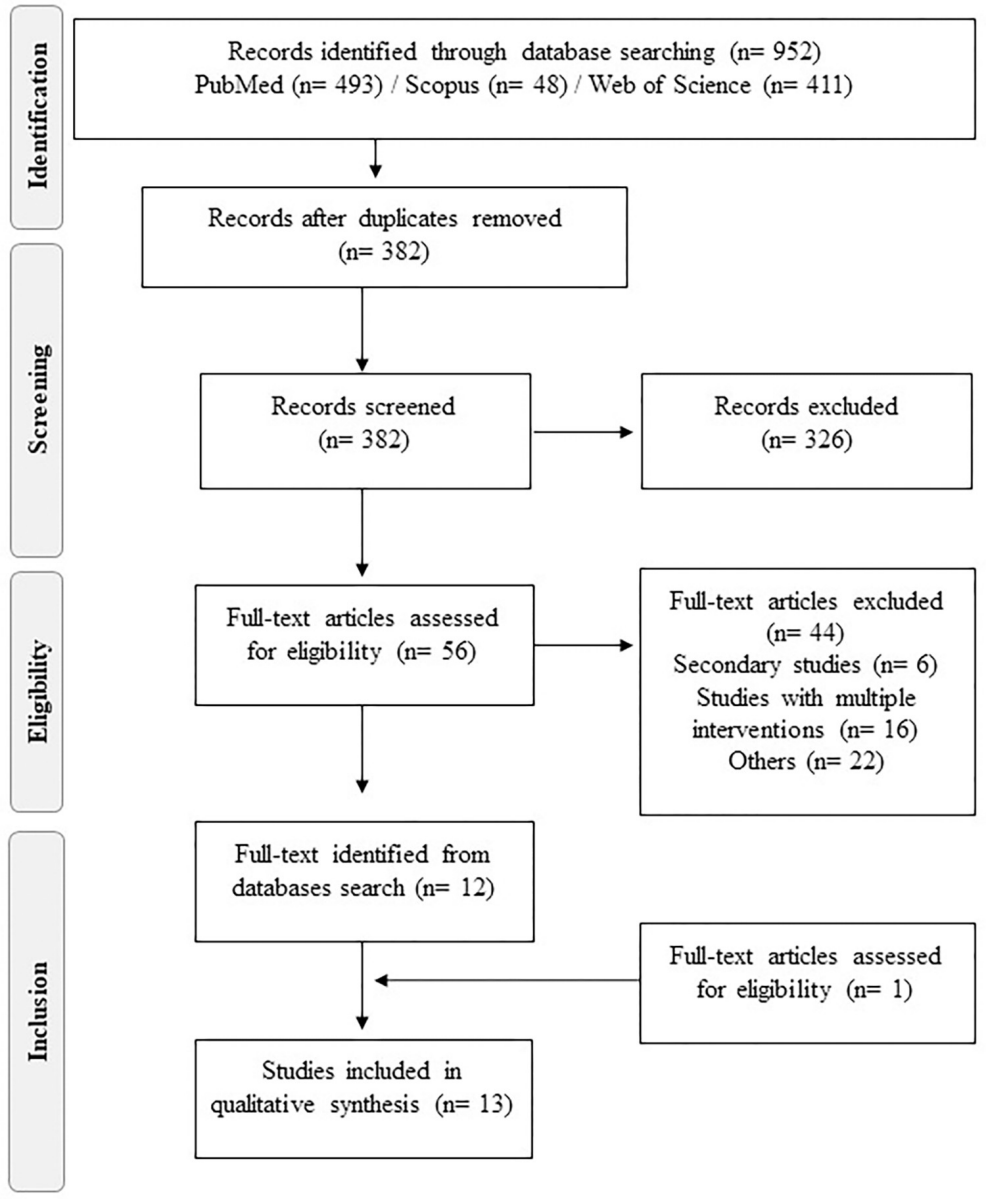
Flowchart detailing selection of studies included in systematic review. Based on PRISMA statement “Preferred Reporting Items for Systematic Reviews and Meta-Analyses”; www.prisma-statement.org.

All animal (in vivo) and human original studies that evaluated the TCR repertoire on Chagas disease were included in this systematic review. The guiding question constructed to determine the inclusion criteria was set according to the PICO (Population, Intervention, Comparison, Outcome, and Study design) strategy. The duplicates of the Scopus and Web of Science database were removed manually through comparison of the authors, title, year, volume, edition, and publication journal [29]. At first, the title, summary, and keywords of all studies were analyzed, and irrelevant searches were excluded. Then, the remaining papers were collected, and the texts were read in full, analyzing the eligibility according to the exclusion and inclusion criteria. Inclusion criteria were in vivo preclinical and human original studies that evaluated TCR repertoire on Chagas disease. The exclusion criteria were as follows: (i) in vitro studies; (ii) studies that analyzed B cell receptor repertoire or TCR repertoire in diseases caused by other trypanosomatids (the focus of this systematic review was TCR repertoire in *T*. *cruzi* infection); (iii) descriptive studies, for example, annals of congresses, letters, case reports, reviews, and editorials (these studies may provide analyzes with incomplete methodology, thus making it difficult to reach a strong conclusion); (iv) papers that analyzed multiple interventions (such as the use of drugs to other diseases, medicinal plants, among other interventions) that can change the frequency, TCR repertoire or that does not allow evaluating the TCR repertoire without interventions; (v) full text unavailable; and (vi) gray literature (studies not indexed and not submitted to the formal peer review process) [27]. The eligibility of the studies was performed independently by the reviewers, and the disagreements were resolved between them (T.G.S.S., K.J.G., and W.O.D.). The flowchart adopted to select the studies is presented in Fig 1.

### Data extraction

The data extraction of each study was performed considering a detailed examination of all papers, based on methodological rules described for preclinical studies [28]. For all studies relevant to this review, data from the publication were collected such as authors, publication year, and country. In animal models studies, the following information has been extracted: (i) animal lineage; (ii) sex; (iii) age; (iv) *T*. *cruzi* strain; (v) *T*. *cruzi* inoculum size; (vi) infection time; and (vii) technique for analyzing the TCR repertoire. Additionally, the primary outcomes for the in vivo preclinical studies were parasitemia and mortality, while the secondary outcomes were data regarding TCR variable regions preferably expressed, immunological markers, and histopathological findings. For the human studies, the following information has been collected: (i) sex; (ii) age; (iii) diagnostic methods of Chagas diseas; (iv) clinical form of the disease; and (v) technique for analyzing the TCR repertoire. The primary outcomes for the human studies were information on parasitemia, mortality, and life quality, while the secondary outcomes were information on TCR variable regions preferably expressed, immunological markers, and cardiac function [29].

### Quality and risk of bias analysis of preclinical studies

The quality of preclinical studies was performed by using the instrument that provides a complete analysis of each section of the paper (title and abstract to acknowledgments and funding). The tool “Animal Research: Reporting of In Vivo Experiments” (ARRIVE) was developed according to basic guidelines recommended for animal research [30], leading consideration essential requirements that should be reported in studies with animals’ models.

The risk of bias in studies with animal models was evaluated by according the tool SYRCLE’s (Systematic Review Centre for Laboratory animal Experimentation) [31], which was developed based on Cochrane Collaboration (RoB 2.0), and it proposes to assess in an adjusted way for aspects of bias that play an important role in animal intervention studies. SYRCLE’s tools were structured into 10 topics, based on the areas of the following: (i) randomization; (ii) basic characteristics; (iii) allocation concealment; (iv) random housing; (v) evaluator blinding; (vi) blinding of outcome evaluator; (vii) blinding of outcomes; (viii) incomplete information; (ix) selective finding reporting; and (x) ethical considerations [31].

### Quality and risk of bias analysis of human studies

The potential risk of bias in human studies was evaluated by using “Downs and Black Measuring Quality.” For this analysis, 27 questions were structured in the following categories: (i) relate quality; (ii) validity; (iii) bias; (iv) confounding; and (v) statistical analysis [32,33].

## Results

### Included studies

The initial search retrieved 952 studies in the 3 datasets, PubMed, Scopus, and Web of Science. However, after removing duplicate studies (*n =* 560), a total of 382 papers were screened for title and abstract, in order to exclude irrelevant studies. The studies that remained after the identification and screening stages (*n =* 56) were read in the full for application of inclusion and exclusion criteria. A total of 13 studies, 3 in vivo preclinical studies and 6 human studies, were included in this systematic review (Fig 1).

### General characteristics of in vivo preclinical studies of T-cell repertoire in *Trypanosoma cruzi* infection

Seven studies evaluating the TCR repertoire on animal models of *T*. *cruzi* infection were retrieved from the 2 search strategies. All the studies used mice as animal model, with age ranging from 6 to 10 weeks. Only isogenic animals were used in the preclinical studies, mainly male mice (*n =* 4; 57%) and 2 papers did not report the sex of the animals. The *T*. *cruzi* strains used were as follows: CL (*n =* 2; 28.6%), Tulahuén (*n =* 2; 28.6%), Colombian (*n =* 1; 14%), CA-I (*n =* 2; 28.6%), and RA (*n =* 1, 14%). Most of the studies used *T*. *cruzi-*TcVI genotypes (*n =* 5; 71.4%), one report used TcI (14%), and Tekiel and colleagues [34] used TcI and TcVI genotypes. The different inoculation routes used were intraperitoneal (*n =* 5; 71.4%) and intradermoplantar (*n =* 2; 28.6%). The inoculum size was heterogeneous among the studies. One study evaluated exclusively the acute phase of the infection (14.4%), while 3 papers evaluated the chronic phase (42.8%) and the other 3 simultaneously reported data from acute and chronic infection. Flow cytometry was the most used technique to assess the TCR repertoire (*n =* 5; 71.4%), while 2 other studies used reverse transcription PCR (RT-PCR) (Table 1).

**Table 1:** General characteristics of studies with preclinical models of *T*. *cruzi* infection.

Author	Country	Animal lineage	Age	Sex	*T*. *cruzi* strain	Route of inoculation	*T*. *cruzi* inoculum*	Infection time	TCR Tech.**
Leite-de-Moraes et al., 1994 [39]	France	C57BL/6, C3H/HeJ, and BALB/c	6–8 weeks	(-)	CL	Intrap.	1 ×10^5^	7–14 days and >180 days	Flow cytometry
Cordeiro Silva et al., 1996 [36]	France	BALB.*xid*	7–8 weeks	(-)	CL	Intrap.	1 ×10^4^	15 days	Flow cytometry
Cardoni et al., 1996 [37]	Argentina	BALB/c, CBA/HJ, and CBA/J	9 weeks	M	Tulahuén	Intrap.	50	21 days and 14 weeks	Flow cytometry
Sunnemark et al., 1998 [38]	Sweden	CBA/HJ and Balb/cB	8–10 weeks	F	Tulahuén	Intrap.	50	9 months	RT-PCR
Mendes-da-Cruz et al., 2003 [40]	Brazil	BALB/c	6–9 weeks	M	Colombian	Intrap.	1 ×10^5^ or 1 × 10^2^	3 weeks and 4–5 months	Flow cytometry
Tekiel et al., 2005 [34]	Argentina	C3H/HeN	8 weeks	M	RA or CA-I	RA: Intrad. CA-I: Intrap.	10–30 (RA strain) or 1 ×10^5^ (CA-I strain)	4 months	RT-PCR
Vogt et al., 2008 [41]	Argentina	C3H/HeN	8 weeks	M	CA-I	Intrad.	100	6 months	Flow cytometry

(-) Data not investigated or reported.

*Number of trypomastigotes inoculated in each animal.

**TCR Tech., TCR repertoire evaluation technique.

F, Female; Intrad., Intradermoplantar; Intrap., Intraperitoneal; M, Male; RT-PCR, reverse transcription PCR.

### General characteristics of human T-cell repertoire studies during Chagas disease

We found 6 human studies, which evaluated TCR repertoire in Chagas patients from Bolivia (*n =* 2, 33.3%), Venezuela (*n =* 1, 16.7%), and Brazil (*n =* 3, 50.0%). None of them reported the sex of the population studied, and only 2 studies evaluated the TCR repertoire in Chagas children and newborns [24,35]. All the studies used at least 2 methods for the diagnosis of *T*. *cruzi* infection, mostly serologic. Electrocardiography methods were used to determine the clinical forms. Flow cytometry was the technique mostly used to evaluate TCR repertoire in those studies (Table 2).

**Table 2:** General characteristics of human studies with Chagas patients.

Author	Sex/Country	Age (years)	Diagnostic of ChD	Clinical form of the disease	TCR repertoire evaluation technique
Cunha-Neto et al., 1994 [42]	ND/Brazil	(-)	Serology and ECHO	Dilated cardiomyopathy and severe heart failure	RT-PCR
Costa et al., 2000 [24]	ND/Bolivia and Brazil	Children and 32–54 years	Serology, radiology, and ECG	Asymptomatic acute and chronic	Flow cytometry
Fernández et al., 2002 [43]	ND/Venezuela	44–81	Serology and ECG	Asymptomatic, arrhythmia and congestive heart failure	RT-PCR
Hermann et al., 2002 [35]	ND/Bolivia	Newborns	Hemoculture and microscopic examination*	(-)	Flow cytometry
Menezes et al., 2004 [44]	ND/Brazil	26–61	Serology and ECG	Indeterminate, nondilated cardiopathy and dilated cardiopathy	Flow cytometry
Menezes et al., 2012 [25]	ND/Brazil	27–75	Serology, ECG, ECHO, and radiological evaluation	Indeterminate and dilated cardiomyopathy	Flow cytometry

(-) Data not reported or investigated.

ChD, Chagas disease; ECHO, echocardiography; ECG, electrocardiography; ND, Not determined; RT-PCR, reverse transcription PCR; TCR, T-cell receptor.

*Microscopic examination of heparinized microhematocrit tubes.

### TCR repertoire in experimental Chagas disease

Only 2 studies evaluated the parasitemia of the animals. BALB.*xid* mice from the original breeding colony presented a reduction of the parasitemia, high frequency of Vβ6 T-cell producing IFN-γ and resisted to infection compared to BALB.*xid* mice adoptive fostering. The latter showed 100% mortality 2 weeks after infection. In addition, BALB.*xid* mice became susceptible to infection and displayed reduced ability to produce IFN-γ after blocking TCR Vβ6 [36] (Table 3).

**Table 3:** Primary and secondary outcomes of in vivo preclinical studies of *Trypanosoma cruzi* infection.

Author/ Date	Primary outcomes	Secondary outcomes
Leite-de-Moraes et al. 1994 [39]	Decreased: CD4^+^ splenic T cells in acutely infected C57BL/6 Increased: Total CD8^+^ splenic T cells in acutely infected C3H/HeJ and BALB/c (I-E^+^)	*Acute phase infected C57BL/6*: No alteration of Vβ CD4^+^ cells repertoire Decreased: CD8^+^ splenic T cells expressing Vβ8.1+8.2 Increased: % of Vβ5 and Vβ14 CD8^+^ splenic T cells *Chronic phase infected-C57BL/6*:The distribution of Vβ families were similar to noninfected mice*Acute phase infected C3H/HeJ and BALB/c (I-E*^*+*^*)*:Increased: Vβ5 (double), Vβ6, Vβ14 (except BALB/c) CD8^+^ spleen T cellsDecreased: Vβ8.1 and 8.2 CD8^+^ spleen T cells
Cordeiro Silva et al. 1996 [36]	*Adoptive BALB*.*xid*: *S*usceptible to the infection, 100% mortality after 2 weeks *BALB*.*xid from original breeding colony*: Decreased: Parasitemia, resisted to infection Increase: *Total* CD4^+^ and CD8^+^ splenic cells	*BALB*.*xid from original breeding colony*: Increased: -*Vβ6* and Vβ8.1+8.2 CD4^+^ cells and Vβ6, Vβ8.1+8.2 Vβ14 CD8^+^ cells in the spleen and IFN-γ -Susceptibility to the infection Decreased: IFN-γ level when blocking Vβ6
Cardoni et al. 1996 [37]	*Acutely infected BALB/c*: Peak of the parasitemia at 21 d.a.i, Decreased: -Cellularity and size of the thymus and % of the CD4^+^CD8^+^ thymocytes Increased: CD4^+^ and CD8^+^ single-positive thymocytes *CBA/HJ and CBA/J mice*: Peak of parasitemia at 4 w.a.i. with alterations of the thymocytes similar to BALB/c mice	*Acutely infected BALB/c*: Increased: -Vβ2, 4, 6, 8, 10, and 14 CD4^+^ thymocytes -Vβ2, 4, 6, 7, 8, and 9 CD8^+^ thymocytes and Vβ6 CD8^+^ splenic cells Decreased: Vβ3, 5, 9, 11, and 12 CD4^+^ thymocytes *Acutely infected CBA/HJ mice*: Increased: Vβ2, 8 and 10 CD4^+^ and Vβ8 CD8^+^ thymocytes Decreased: Vβ8 CD8^+^ splenic cells *Acutely infected CBA/J mice*: Decreased: Vβ8 CD4^+^ and CD8^+^ cells and increased Vβ14 CD4^+^ cells in the spleen
Sunnemark et al. 1998 [38]	No identification of the parasite on the heart or spleen tissue *Chronically infected CBA/HJ*: Increased: CD4^+^ and CD8^+^ T cells in the cardiac tissue	No difference in the usage of the Vβ TCR repertoire between CBA/HJ and Balb/cB mice *Vβ8S2 and 8S3 of the CBA/HJ mice*: Increased: -Vβ8S2 and 8S3 T cells in the heart with non-Gaussian CDR3 length profile -Vβ8S2 and 8S3 splenic CBA/HJ showed a CDR3 length profile similar to noninfected mice
Mendes-da-Cruz et al. 2003[40]	*Acutely infected BALB/c*: Decreased thymic cellularity and there was thymic atrophy Increased: -SP and DN thymocytes -DN, DP, and SP cells expressing CD69^high^CD62L^low^ in the lymph node *Chronically infected BALB/c*: Increased: cellularity in the LN and CD4+ T cells subsets	*Acutely infected BALB/c*: Increased: -Vβ12 and Vβ8 CD4^+^ and Vβ8 CD8^+^, Vβ8^+^CD25^+^ DP cells in the LN -Vβ5 and Vβ12 DP cells and CD4^+^T cells expressing CD62L in the thymus *Chronically infected BALB/c*: Increased: Vβ5 and Vβ8 DP cells, Vβ8 CD4^+^ and Vβ12 CD8^+^ cells in the LN
Tekiel et al. 2005 [34]	(-)	*Infected C3H/HeN with RA strain*: Increased: -Vβ1, 8.1, 8.2, 10, and 13 (polyclonally) T cells in the spleen -Vβ8.1 T cells in the skeletal muscle -Vβ1, 8.1, 8.2, 9 (oligoclonality), 10, and 13 (polyclonally) T cells in the spinal cord -Vβ8.2 and 9 T cells in the sciatic nerve Decreased: -Vβ14 T cells in the spleen -Vβ6, 11, 14 e 16 T cells in the skeletal muscle -Vβ7 T cells in the spinal cord -Vβ11 and 14 T cells in the sciatic nerve *Infected C3H/HeN with CA-I strain*: Increased: -Vβ4, 6, 13, 15, and 16 T cells in the spleen -Vβ9 (polyclonally) and 16 T cells in the skeletal muscle -Vβ4 T cells in the spinal cord Decreased: -Vβ14 T cells in the spleen with polyclonally in Vβ9 T cells (frequency unchanged) -Vβ15 T cells in the skeletal muscle -Vβ11 and 12 T cells in the spinal cord
Vogt et al. 2008 [41]	(-)	Increased: % Vβ9 CD4 and CD8 T cells in the skeletal muscle and heart tissue Decreased: -Vβ 6, 8.1, 8.2, 9, 15, and 17 in the sciatic nerve -Vβ9 T cells from skeletal muscle and heart tissue show higher response to amastigote antigen and produce IL-10 - Vβ9 T cells from lymph node or nonlymphoid do not respond to the self-antigen -Vβ9 T cells from lymph node secreted IFN-γ under stimulation with amastigote antigen

(-) Data not investigated or reported.

d.a.i., days after infection; DN, double-negative; DP, double-positive; LN, lymph node; SP, single-positive; w.a.i., weeks after infection.

Acutely infected BALB/c mice presented a peak of parasitemia 21 days after infection, while CBA/HJ and CBA/J mice showed a peak of parasitemia 4 weeks after infection [37]. The parasite burden was evaluated in only one study, and parasites were not found in the heart and spleen of the CBA/HJ and Balb/cB mice after 9 months of the infection [38] (Table 3). In *T*. *cruzi*-resistant C57BL/6 mice, acute infection decreased CD4^+^ T cells in the spleen [39], while in BALB.*xid* and BALB/c mice, the acute infection increased CD4^+^ and CD8^+^ T cells in the spleen [36]. It also increased single-positive and double-negative thymocytes and increased single-positive, double-negative, and double-positive T cells expressing CD69^high^ and CD62^low^ markers in the lymph node [40]. Thymic atrophy and reduction of the thymic cellularity in *T*. *cruzi*-infected acutely BALB/c mice was also observed [37,40]. The chronically *T*. *cruzi*-infected CBA/HJ mice showed that the CD4+ and CD8+ T cell infiltrate increased in the cardiac tissue [38] (Table 3).

The acute infection of C57BL/6 mice by *T*. *cruzi* did not alter the CD4+ T-cell TCR repertoire but increased the frequencies of splenic CD8+ cells expressing Vβ5 and Vβ14 and decreased the frequencies of splenic CD8^+^ cells expressing Vβ8.1 and Vβ8.2. When analyzing the TCR repertoire in C3H/HeJ and BALB/c mice acutely infected with *T*. *cruzi*, the same Vβ families were altered in CD8^+^ splenic cells, but the distribution of the other Vβ families was similar to the noninfected animals [39]. In BALB/c, CBA/HJ, and CBA/J mice, the acute infection by *T*. *cruzi* increased the frequency of Vβ2, 4, 6, 8, 10, and 14 CD4^+^ thymocytes, Vβ14 CD4^+^ spleen cells, and Vβ12 CD4^+^ T cells in the lymph node. On the other hand, a decrease of the Vβ3, 5, 9, 11, and 12 CD4^+^ thymocytes and Vβ8 CD4^+^ spleen cells was observed [37,40]. Regarding CD8^+^ cells, the acute infection by *T*. *cruzi* increased Vβ2, 4, 6, 7, 8, and 9 thymocytes, Vβ8 T cells in the lymph node, and Vβ5, 6, and 14 spleen cells, while Vβ8.1 and 8.2 were decreased in the spleen [37,39,40]. Additionally, Sunnemark and colleagues did not observe differences in the use of Vβ families of the TCR between BALB/c and CBA/HJ mice infected by *T*. *cruzi*. However, when analyzing the T-cell infiltrate in the cardiac tissue of CBA/HJ mice, they found an increased frequency of T cells expressing Vβ8S1 and Vβ8S2. The latter cell population showed a non-Gaussian CDR3 length profile, while Vβ8S1 and Vβ8S T cells of the spleen presented CDR3 length profile similar to noninfected mice [38] (Table 3).

Mendes-da-Cruz and colleagues reported that chronically infected BALB/c mice presented an increased CD4^+^CD8^+^ T cells bearing Vβ5 or Vβ8, Vβ8 CD4+, and Vβ12 CD8+ T cells in the lymph node. Chronic infection by a highly virulent *T*. *cruzi* strain (RA strain, TcVI) resulted in changes in the TCR repertoire in different organs. An increased frequency of Vβ1, 8.1, 8.2, 10, and 13 T cells, and a decreased frequency of Vβ14 T cells in the splenic tissue were observed. On the other hand, an increase of Vβ8.1 T cells and a decrease of Vβ6, 11, 14, and 16 T cells were observed in skeletal muscle. Vβ1, 8.1, 8.2, 9, 10, and 13 T cells were increased, and Vβ7 T cells were decreased in spinal cord. Finally, Vβ8.2 and Vβ9 T cells were increased, and Vβ11 and 14 T cells were decreased in sciatic nerve [34].

These same authors used the same mice lineage (C3H/HeN), infected with a low virulence *T*. *cruzi* strain (CA-I strain, TcI) and also analyzed the TCR repertoire in different tissues in the infection chronic phase. They observed an increase of Vβ4, 6, 13, 15, and 16 spleen cells and a decrease of Vβ14 spleen cells. In the skeletal muscle, the frequency of Vβ9 and 16 T cells was increased, while the frequency of Vβ15 T cells was decreased. Vβ4 T cells were increased, and Vβ11 and Vβ12 T cells were decreased in the spinal cord, and there was a decrease in Vβ6, 8.1, 8.2, 9, 15, and 17 T cells in sciatic nerve [34]. Interestingly, in the cardiac and skeletal muscles, an increase of CD4^+^ and CD8^+^ T-lymphocytes expressing Vβ9 TCR was observed, and these cells presented a greater response once stimulated with amastigote antigen and produced the regulatory cytokine IL-10. Vβ9 T cells of the lymph node did not respond to self-antigen, but they secreted IFN-γ when stimulated with amastigote antigen [41] (Table 3).

### TCR repertoire in human Chagas disease

Children with acute asymptomatic Chagas disease presented a lower frequency of CD4^+^ T cells expressing Vβ5 TCR and normal distribution of the Vβ2, 3.1, 8, and 17-expresing CD4^+^ T cells [24]. In addition, the showed normal levels of Vβ2, 3.1, 5, 8.1, and 17-expressing CD8^+^ T cells in the peripheral blood [24]. In addition, there was a reduction in the frequency of CD4^+^ T cells expressing Vβ5, 11, 13.1, and 18, while there was an increase of Vβ5, 13.1, 16, 17, and 22 in CD8^+^ T cells, single-positive T cells (CD4^+^ and CD8^+^) producing IFN-γ, TNF-α, and perforin molecules [35] (Table 4).

**Table 4:** Primary and secondary outcomes of human studies with Chagas patients.

Author/Date	Infection phase	Primary outcomes	Secondary outcomes
Cunha-Neto et al. 1994 [42]	Chronic	(-)	-Heart tissue showed signs myocarditis and destruction of cardiac myofibrils associated to diffuse lymphocyte infiltrate -Decreased: number of the Vα transcripts than Vβ transcripts -Increased: cells expressing Vα13 and Vα2 TCR -Cells of the heart tissue expressed Vβ1, 2, 3, 4, 5.1, 5.2, 6, 7, 8, 9, 10, 11, 12, 14, 15, and 18 transcripts
Costa et al. 2000 [24]	Acute and chronic	(-)	*Chagas children with an asymptomatic acute phase*: Decreased: Vβ5 CD4^+^ T cells and normal distribution of the Vβ2, 3.1, 8, and 17 CD4^+^ T cells and Vβ2, 3.1, 5, 8.1, and 17 CD8^+^ T cells *Chronic Chagas adults*: -Vβ5 CD4^+^ T cells in IND are similar to uninfected Increased: -Vβ17 CD4^+^ T cells from IND when stimulated with TRYP -Vβ5 CD4^+^ T cells in CCC patients -Expansion of the Vβ5 CD4^+^ and CD8^+^ T cells from IND or CCC patients after stimulation with TRYP or EPI -Expansion of Vβ5 CD4^+^ and Vβ17 CD8^+^ T cells from CCC when stimulated with TRYP
Fernández et al., 2002 [43]	Chronic	(-)	Increased: -Vβ4, 5, 7, 11, 13, 17, and 20 T cells in symptomatic patients -Vβ4, 5, 11, 13, 17, and 20 T cells in HF patients -Vβ4, 5, 9, 11, 13, 17, and 19 T cells in asymptomatic patients and TCR repertoire more restrict -Vβ4, 5, 7(mainly), 11, 17, and 18 in arrhythmic patients and TCR repertoire more restrict -Vβ7 T cells in DRB1*01, DQB1*0501, and DPB1*0401 haplotype patient with arrhythmia or CHF Decreased: Vβ2, 3, 6, 14, 15, and 16 T cells in symptomatic patients
Hermann et al. 2002 [35]	*	Increased: *-*CD8^+^CD45RO^+^ and CD8^+^HLA-DR^+^ T cells -CD4^+^CD45RO+HLA-DR^+^ T cells -CD8^+^ T cells expressing CD28 molecule	Increased: -Vβ5, 13.1, 16, 17, and 22 CD8^+^ T cells -Annexin V and apoptosis death by CD8+ and CD4+ T cells -IFN-γ, TNF-α, and perforin producing CD4^+^ and CD8^+^ T cells -IL-2- or IL-4 producing T cells similar to non-Chagas newborns Decreased: -Expansion of the Vβ5, 11, 13.1, and 18 CD4^+^ T cells
Menezes et al. 2004 [44]	Chronic	(-)	Increased: -Vβ5^+^CD4^+^CD28^+^, Vβ5^+^CD4^+^CD28^−^, and Vβ3.1^+^CD8^+^CD28^+^ T cells from IND, DC, and NDC patients -Vβ5^+^ and Vβ3.1^+^ CD8^+^CD28^+^ and Vβ5 and Vβ8 CD4^+^CD28^+^T cells in IND patients Decreased: -Vβ3.1^+^CD4^+^CD28^+^ T cells and increase IFN-γ, TNF-α, and IL-4 cytokines in DC and NDC patients -Vβ2, 8 and 17 CD8^+^CD28^−^ T cells in CCC -There were association frequencies CD8^+^CD28^+^ and TNF-α or IFN-γ producing T cells in CCC and association frequencies CD4^+^IL-10 and CD4^+^CD28^−^ or CD4^+^CD28^+^ in IND
Menezes et al. 2012 [25]	Chronic	(-)	Increased: -Vβ5.2 CD4^+^T cells in CCC -Vβ5.1 and 5.2 CD4+ T cells in IND Decreased: -Vβ9 CD4^+^ T cells in CCC -Vβ9 CD4+ T cells in IND -Vβ5.1^+^CD4^+^ T cells from CCC and 1 IND showed homologous CDR3 motif: Maintenance of CDR3 size, BJ gene segment conservation, and conservation of amino acids usage at positions 94, 95, 97, and 98 of the TCR-β-chain. -Association CD4^+^Vβ5.1 and CD4^+^GranzimaA^+^ T cells in CCC

(-) Data not investigated or related.

*Chagas newborns.

CCC, chronic Chagas cardiomyopathy; DC, dilated cardiopathy; EPI, epimastigote; HF, heart failure; IND, indeterminate; NDC, nondilated cardiopathy; TCR, T-cell receptor; TRYP, trypomastigote.

Tissue myocarditis was reported in chronic Chagas patients, accompanied by destruction of cardiac myofibrils [42]. The study by Cunha-Neto and colleagues was the only study to evaluate the Vα TCR repertoire in Chagas disease. They observed an increase in Vα2 and Vα13 TCR, while T cells expressing Vβ1, 2, 3, 4, 5.1, 5.2, 6, 7, 8, 9, 10, 11, 12, 14, 15, and 18 were overrepresented in cardiac tissue of chronic Chagas patients [42] (Table 4).

Analyzing the TCR repertoire of peripheral blood T lymphocytes from symptomatic chronic Chagas patients from Venezuela, Fernandez-Mestre and colleagues [43] reported a reduction in the frequency of total T cells expressing Vβ2, 3, 6, 14, 15, and 16. Regarding cell subpopulations, CCC patients from Brazil presented an increase of Vβ5.2-expressing CD4^+^ and Vβ17-expressing CD8^+^ T cells, but also a reduction of Vβ9-expressing CD4^+^ T cells [24,25]. Interestingly, the CD4^+^ T cells of CCC patients expressed DRB1*13 and DERAA motif in the positions 70 to 74 [25], and those increased T cells bearing Vβ7 TCR presented haplotype DRB1*01, DQB1*0501, and DPB1*0401 [43]. Additionally, there was an association of frequency CD8^+^CD28^+^ T cells with the production of TNF-α and IFN-γ cytokines, and an association of Vβ5.1 CD4^+^ T cells with CD4^+^GranzymA^+^ in CCC [25,44] (Table 4).

According to the studies, patients with the indeterminate clinical form showed an increase in the frequency of Vβ3.1, 5, and 8 expression in CD4^+^CD28^+^ cells, Vβ5 in CD4^+^CD28^−^ cells, Vβ3.1 in CD8^+^CD28^+^ T cells, while Vβ9 was down-represented in CD4^+^ T cell [25,44]. In addition, the TCR repertoire of asymptomatic patients seems to be more restricted, with an increase of Vβ 4, 5, 9, 11, 13, 17, and 19-expressing T cells [43] (Table 4).

### Reporting quality of preclinical and human studies

**Fig 2:**
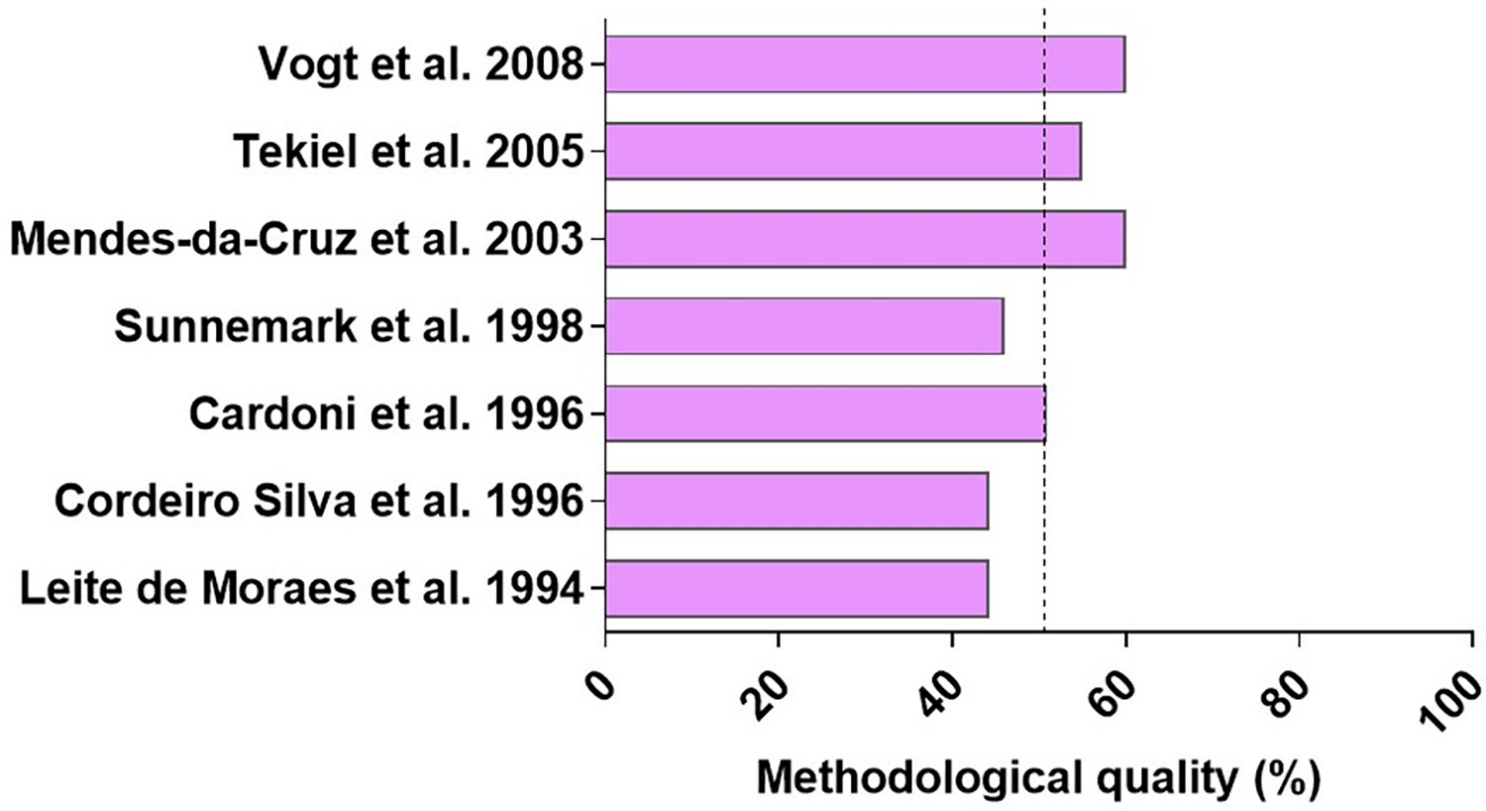
Reporting quality in preclinical studies evaluating which variable regions of TCR were preferentially expressed on in vivo models of *Trypanosoma cruzi* infection. The dotted line indicates the mean methodological score (%). ARRIVE, Animal Research: Reporting of In Vivo Experiments; TCR, T-cell receptor. References: [34,36–41].

Analyzing the quality of the studies included in this systematic review, about 51.48% of quality criteria were completed by the in vivo preclinical studies. The animal model studies performed up to 20 years ago, like Leite-de-Moraes and colleagues and Cordeiro and colleagues, were those that showed the greatest gap in the details and methodological adequacy (Fig 2).

**Fig 3:**
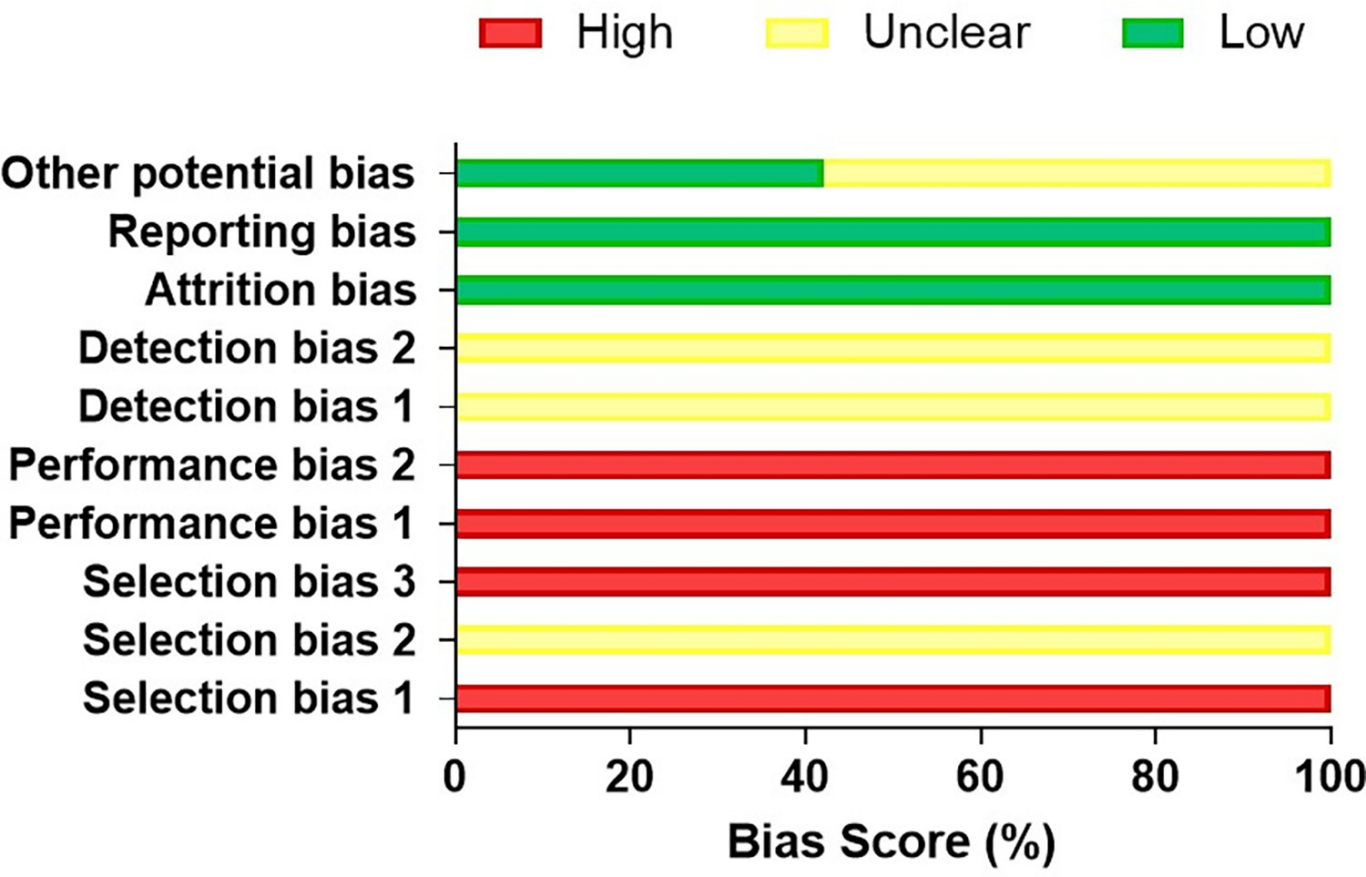
Rating of the animal studies using the SYRCLE’s risk of bias toll for animal studies (BMC Medical Research Methodology 14:43, 2014).

The main criteria that have not been properly met were related to the ethical statement, study design, housing and husbandry, baseline data, and generalizability/translation, which can be checked in S2 Table. Additionally, the SYRCLE’s tool indicates that the main risk of bias in animal studies was related to allocation sequence applied, randomization strategy, and blinding of caregivers and/or investigators. The lowest risk of bias was related to adequacy of the treatment of incomplete data and free of selective outcome (S3 Table and Fig 3).

**Fig 4:**
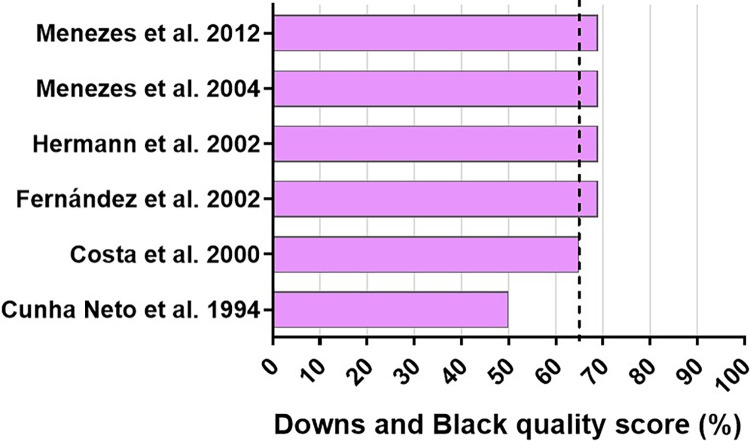
Reporting quality in human studies evaluating which variable regions of TCR were preferentially expressed on *Trypanosoma cruzi* infection. The dotted line indicates the mean methodological score (%). Downs and Black Measuring Quality in randomized and nonrandomized clinical assays (Journal of Epidemiology and Community Health 52:377–384, 1998). References: [24,25,35,42–44].

As in preclinical animal studies, no human study met all quality criteria according to Downs and Black checklist. The average quality criteria completed by human studies was a score of 65.16%, and the study that presented the highest risk of bias was performed by Cunha-Neto and colleagues. In general, the limitations of human studies were related to blinding of measuring the main outcomes, characteristics of patients lost to follow-up, and randomized intervention from patients and staff (S4 Table and Fig 4).

## Discussion

From a comprehensive analysis of a limited number of studies, the data indicate that the despite the great variability in the usage of TCR Vβ regions, there is a down-regulation of TCR Vβ5^+^ expression by T cells in the acute phase of the Chagas disease [24,35,44], but this region apparently is overly expressed by T cells in the chronic phase of the disease [25,44]. Interestingly, the outcomes suggest that while Vβ9^+^ T cells had a modulatory profile in muscle tissue, these same cells produce inflammatory cytokines when in lymphoid organs [41].

### In vivo preclinical studies

All in vivo preclinical studies employed young and isogenic mice to evaluate the TCR repertoire in experimental infection by *T*. *cruzi*, which are important factors in the determination of resistance or susceptibility to infection [45,46], as well as greater control of immunological variability [47]. Clearly, there are isogenic animals that may or may not establish a rapid, vigorous, and effective immune response in dealing with the parasite, which are classified as resistant or susceptible animals to infection by *T*. *cruzi* [48]. Additionally, in order to reproduce the pathophysiological characteristics well described in human Chagas disease, the choice of lineage should be based on the phase of infection (acute or chronic), virulence of the *T*. *cruzi* strain [49,50], and size of the inoculum [47]. The virulent strains of *T*. *cruzi* (Colombian, Tulahuén, CL, and RA strain) were used to infect susceptible mice BALB/c, CBA/HJ, and C3H/HeJ lineage [34,36–40]. On the other hand, the less virulent strain (CA-I strain) was used in susceptible mice C3H/HeN and BALB/c. In both cases, analysis was performed during chronic infection [34,41].

In the acute experimental *T*. *cruzi* infection, the outcomes confirmed a polyclonal activation of CD4^+^ and CD8^+^ T cells in the spleen, lymph node, and thymus of resistant and susceptible mice. This polyclonal response suggests that lymphocytes may have been activated by mechanisms that depend on the recognition of a broad range of *T*. *cruzi* antigens and possibly other stimuli [51]. While the effector function of different classes of T lymphocytes is crucial for controlling infection, this nonspecific response can have a crucial role in pathology establishment [51]. Despite this complex immune response, the analysis of the TCR variable region revealed a high frequency of CD8^+^ T lymphocytes expressing Vβ5, 6, 14, 8.1, and 8.2 in lymphoid organs such as spleen and thymus [37–40].

The fact that there is an overrepresentation of CD8^+^ T cells expressing variable regions Vβ5, Vβ6, Vβ8, and Vβ14 in acute infection suggests that these cells were stimulated by a dominant antigen and that they may influence infection outcome [37,39,40]. Corroborating this possibility, Cordeiro da Silva and colleagues showed that blocking T cells bearing Vβ6 TCR rendered BALB.*xid* mice susceptible to infection, associated with an expressive reduction in IFN-γ levels. These data emphasized the importance of these Vβ6 T cells in infection control by a mechanism clearly involving the production of an inflammatory cytokine [52,53].

Experimental studies that evaluated the chronic phase of *T*. *cruzi* infection did not assess parasitemia, since at this stage of infection the identification of the trypomastigotes is difficult [16]. Thus, it is important to assess the parasitic burden in organs, taking under consideration the tropism of each strain of *T*. *cruzi*, to confirm maintenance of infection.

**Fig 5:**
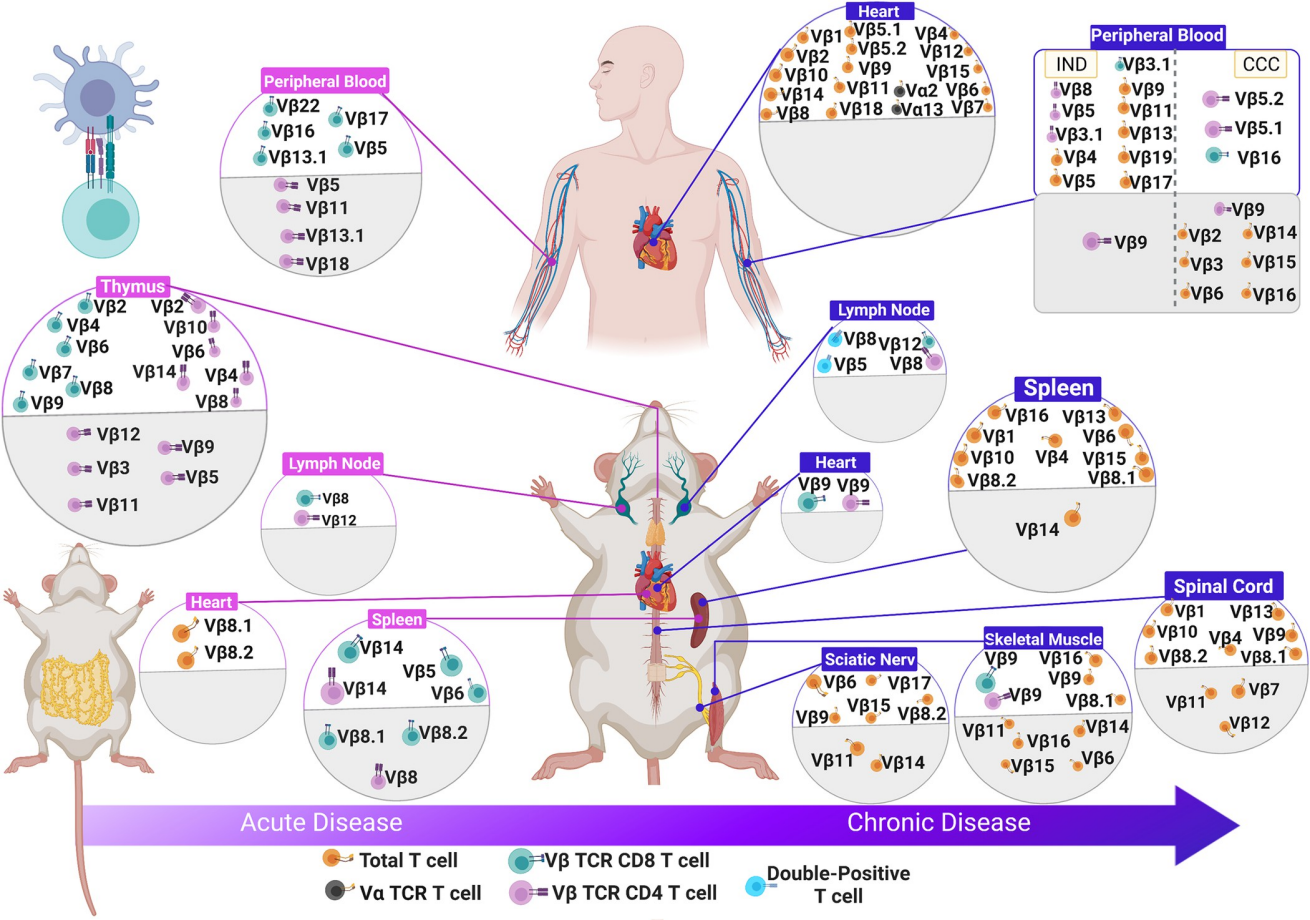
Schematic representation of variable TCR Vβ regions differentially expressed in acute and chronic phase of *Trypanosoma cruzi* infection in murine models, as well as human Chagas disease. The TCRVβ in the white area were up-regulated, while TCRVβ in the gray area were down-represented. CCC, chronic Chagas cardiopathy; IND, indeterminate; TCR, T-cell receptor; Vα, α-chain variable region; Vβ, β-chain variable region. Designed using Biorender.

In the late infection, studies using unseparated CD4^+^ and CD8^+^ T cells showed a reduction in the frequency of T cells expressing Vβ14 in the spleen and in nonlymphoid organs such as skeletal muscle and spinal cord, while the frequency of T cells expressing Vβ6 and Vβ8.1, 8.2 was elevated in the spleen and lymph node [34,40] (Fig 5).

Interestingly, CD4^+^ and CD8^+^ T cells–bearing Vβ9 are increased in the skeletal and cardiac muscle of animals chronically infected with *T*. *cruzi*, and, when stimulated with amastigote antigen, they produced low IFN-γ and IL-4, but high IL-10. On the other hand, Vβ9-TCR T cells derived from lymph nodes stimulated under the same conditions had an inflammatory profile, producing high levels of IFN-γ [41].

### Human studies

Although most studies were performed in chronic adult Chagas patients, we retrieved 2 studies that evaluated the TCR repertoire in children and newborns with Chagas disease [24,35], which is very relevant to expand our understanding of the immunopathology of Chagas disease in different age groups. In children and newborns, the TCR repertoire was similar to the one found in acute and chronic phase of Chagas disease in adults, which was mainly characterized by a decrease of frequency Vβ5 CD4^+^ T cells [24,35].

Regarding methods for diagnosing *T*. *cruzi* infection, with exception of the study that evaluated newborns, all other studies employed serological tests accompanied by a second different method to determine the clinical form of disease. The serological tests, such as indirect immunofluorescence, indirect hemagglutination, and enzyme-linked immunosorbent assays [54], are simple, accessible, and effective for detecting anti-*T*. *cruzi* antibodies in the chronic phase. However, they present limitations regarding sensibility, specificity, and reproducibility [55]. Thus, the World Health Organization advises the use of at least 2 tests with different mechanisms of detection to arrive at a conclusive diagnosis.

Analysis of the studies indicate that there is an expressive modulation of the frequency of T cells bearing TCR Vβ5 variable region, with a decrease in acute phase and increase in chronic phase of Chagas disease [24,35,42]. Cunha-Neto and colleagues were the only authors to evaluate the TCR repertoire in the cardiac tissue of Chagas patients. However, that work did not analyze the TCR from T-cell subpopulations separately, which may have masked possible preferential responses among one of the subpopulations. It is worth noting that this increase in the frequency of TCR Vβ5+ T cells in cardiac tissue [42] reflects what also occurs in the peripheral blood of Chagas patients. Interestingly, Menezes and colleagues observed a positive correlation between the frequency of Vβ5 CD4^+^ T cells and CD4^+^ T cells expressing granzyme A in peripheral blood of Chagas patients, suggestive of cytotoxic function and possibly a role in pathology [56,57]. The role of this cell subpopulation in the progression of Chagas disease, and which antigens, whether parasite antigens, autologous, or superantigens, are responsible for stimulating this family of Vβ5 CD4^+^ T cells in the chronic phase of the infection are important open questions, which go beyond the scope of this systematic review.

Regarding other Vβ families that are preferentially expressed or down-regulated by T lymphocytes from Chagas patients, we found discordant outcomes. The variability in the use of Vβ TCR genes among Chagas patients can be attributed not only to antigenic exposure, but also to differences in HLA (DR or DQ or DP) among the studied populations [58]. This association between Vβ TCR genes and HLA in Chagas disease was explored in the study by Fernandez-Mestre and colleagues, in which they observed that cardiac tissue infiltrating T cells from Chagas patients with arrhythmia presented an increased HLA-DRB2*01 and DQB1*0501 haplotype compared to asymptomatic Chagas patients or those with heart failure. Interestingly, Menezes and colleagues reported that Vβ5 CD4+ T cells were up-regulated in the peripheral blood of CCC patients who expressed the *0103, *0402, *1301, and *1302 alleles of HLA-DRB1, and these alleles are apparently associated with autoreactivity [59].

Unlike experimental studies that did not evaluate the TCR alpha variable region, we retrieved a study that reported the total cardiac tissue infiltrating T cell repertoire using a limited number of TCR Vα subfamilies, Vα2 and Vα13 TCR [42]. This indicates that the TCR Vβ subfamilies in the cardiac lesion of Chagas individuals are less restricted, but more studies are needed to evaluate the alpha chain of the TCR heterodimer. In *Leishmania* infection, a higher variability was observed in TCR Vβ usage, as compared to Vα [60–63]. This more restricted Vα region can be explained by the fact that somatic recombination of the TCR alpha chain occurs only between subregions V (variability) and J (junction).

In vivo preclinical and human studies mainly used flow cytometry or RT-PCR, as tools for investigating TCR variable region usage. Flow cytometry is a rapid, relatively easy method and allows us to characterize in a qualitative and quantitative way the TCR repertoire with specific subpopulations of T cells [64,65]. However, the data generated do not provide information about the composition of the CDR CDR3, which is somatically hypervariable and most closely associated with the recognition of the MHC peptide [13]. Although a panel of 24 monoclonal antibodies for Vβ region are commercially available, some potentially important subfamilies of TCR Vβ may be lost during the analyzes [66]. The RT-PCR molecular approach is a more laborious method that needs ultrapure T cells to ensure accuracy, but in addition to providing a precise and quantitative Vβ and/or Vα TCR repertoire, the sequence of the CDR3 region can be analyzed [67]. Sequencing, more specifically in the CDR3 region, can reveal valuable information on antigen specificity.

### Reporting quality and risk of bias

The assessment of the risk bias potential and the methodological quality of the studies are quality criteria that complement the outcomes reported in the studies. The SYRCLE’s tool highlights as main limitation the allocation sequence and randomization strategy applied to animals, besides if the investigator of analysis was blinded. These limitations raised from the experimental studies, which were evaluated individually, corroborate the high risk of bias in the categories study design, experimental animal, housing and husbandry and baseline data.

Although most human studies are more than 15 years old, they showed better reporting quality compared to experimental studies. However, the inconsistent factors that increase the risk of bias have also been related to methodological design and omission of information. Considering that these factors are easily adjustable, researchers should be encouraged to design methodological strategies based on reproducible and valid guidelines. In the case of studies using experimental animal models, guidelines such as SYRCLE, ARRIVE, and CAMARADES (Collaborative Approach for Meta-Analysis and Review of Animal Data from Experimental Studies) proved information on essential requirements that should be considered and reported in the studies. For human research, guidelines such as SPIRIT (Standard Protocol Items: Recommendations for Interventional Trials) and CONSORT (Consolidated Standards of Reporting Trials) should be consulted to improve methodological quality and minimize the potential risks of bias.

### Limitations and perspectives

This systematic review integrates human and in vivo preclinical studies regarding TCR usage in *T*. *cruzi* infection, and analysis of the immunological profile of T cells bearing specific TCR repertoires. The diversity potential of the human peripheral TCR repertoire is about 10^12^ [68]. Humans evolved with greater diversity of CDR3 compared to mice, and TCR-MHC coevolution may explain the greater diversity of T cells in human [67]. Despite the limitation of comparing the human TCR repertoire to mice, preclinical in vivo studies can provide important insights into the antigenic specificity of rare and potentially relevant clonotypes for the development of diagnostic biomarkers and clinical evolution of Chagas disease.

We found that the TCR repertoire in Chagas disease has been investigated since 1994 in 6 different countries. Most studies originated from endemic countries, such as Argentina, Bolivia, Brazil, and Venezuela, which makes sense given the high frequency, morbidity, and mortality of the disease in these areas. However, we found 2 studies developed in France and Sweden, which are nonendemic countries for Chagas disease, evaluating the TCR repertoire in experimental models of Chagas disease.

A limited number of human and experimental studies assess the TCR repertoire in Chagas disease, and most of these studies date back more than 15 years. The analysis of the TCR repertoire in the context of a pathogen challenge is crucial to understanding basic issues of recognition and immune response [69]. However, this analysis requires specific and laborious protocols and complex analytical tools [70] that have been improved in recent years. This may explain why there was a decline in research using Vβ and Vα TCR family’s analysis in Chagas disease. Furthermore, we observed that studies used flow cytometry or RT-PCR to analyze the TCR repertoire. Flow cytometry is a quick and relatively easy method that allows to qualitatively and quantitatively characterize the TCR [65]. Although a panel of about 24 monoclonal antibodies for determining Vβ regions is currently available, some uncharacterized clonotypes may be lost during the analyzes [66]. With the expansion of the catalogue of anti-Vβ and anti-Vα antibodies, as well as advances in TCR next-generation sequencing techniques, which are more sensitive and accurate in detecting and quantifying rare clonotypes and to decipher the complexity of TCR repertoire, there is a tendency to use more analysis of the TCR repertoire in the context of Chagas disease [70,71]. Of note, the more recent techniques, such as single-cell analysis, are costly, limiting the ability of researchers in countries with restricted funding, which typically happens to be the ones where Chagas disease is endemic, to carry out these studies.

In addition, the TCR repertoire sequencing is useful in understanding the dynamics of T lymphocytes in pathological conditions, such as autoimmune, infectious diseases and cancer [72,73], as well as in identifying TCR biomarkers of response to clinical treatment, immunotherapies, response to vaccination or stratification of patients according to clinical forms [71,74]. In particular, the study of TCR repertoire contributes to understanding the pathogenesis of Chagas disease, but also opens the possibility of developing much needed vaccines and specific immunotherapies against a defined epitope.

## Supporting information

S1 Table Detailed search strategy with search filters and number of studies recovered in electronic databases. (DOCX)

S2 Table Assessment of the reporting quality of the preclinical studies. (DOCX)

S3 Table Evaluation of bias risk in studies with animal models according to the SYRCLE’s tool. (DOCX)

S4 Table Bias analysis of human studies of according to the Downs and Black Quality Index. (DOCX)
