# Supplementary material for: T-cell receptor variable region usage in Chagas disease: A systematic review of experimental and human studies
Source: PLoS Negl Trop Dis. 2022 Sep 15;16(9):e0010546. doi: 10.1371/journal.pntd.0010546 (PMC9477334; doi:10.1371/journal.pntd.0010546)
Supplement: S1 Table — (DOCX) [file pntd.0010546.s001.docx]

**S1 Table. Detailed search strategy with search filters and number of studies recovered in electronic databases.**

| **PubMed-MEDLINE – Search filters** | **Records** |
| --- | --- |
| **#1 Disease model:** (“American trypanosomiasis”[TIAB] OR “South american trypanosomiasis”[TIAB] OR “Trypanosoma cruzi infection”[TIAB] OR “Trypanosoma cruzi”[MeSH Terms] OR “Chagas disease”[MeSH Terms]) | **6.813** |
| **#2 Biological target:** (“T-Cell receptor”[TIAB] OR “T-Cell Receptor alpha”[TIAB] OR “T-Cell Receptor beta”[TIAB] OR “T-Cell Receptor, alpha-beta”[TIAB] OR “T-Cell Antigen receptor specificity”[MeSH Terms] OR “T-Cell receptor specificity”[MeSH Terms]) | **80.176** |
| **#3 Combined search: (**#1 AND #2) | **493** |
| **SCOPUS – Search filters** |  |
| **#1 Disease model:** (TITLE-ABS-KEY(“American trypanosomiasis”) OR TITLE-ABS-KEY(“South american trypanosomiasis”) OR TITLE-ABS-KEY(“Trypanosoma cruzi infection”) OR TITLE-ABS-KEY(“Trypanosoma cruzi”) OR TITLE-ABS-KEY(“Chagas disease”)) | **27.347** |
| **#2 Biological target:** (TITLE-ABS-KEY(“T-Cell receptor”) OR TITLE-ABS-KEY(“T-Cell Receptor alpha”) OR TITLE-ABS-KEY(“T-Cell Receptor beta”) OR TITLE-ABS-KEY(“T-Cell Receptor, alpha-beta”) OR TITLE-ABS-KEY(“T-Cell Antigen receptor specificity”) OR TITLE-ABS-KEY(“T-Cell receptor specificity”)) | **35.881** |
| **#3 Combined search:** #1 AND #2 | **48** |
| **WEB OF SCIENCE – Search filters** | |
| **#1 Disease model:** TS=American trypanosomiasis OR TS=South american trypanosomiasis OR TS=Trypanosoma cruzi infection OR TS=Trypanosoma cruzi OR TS=Chagas disease | **25.289** |
| **#2 Biological target:** TS=T-Cell Receptor OR TS=T-Cell Receptor alpha OR TS=T-Cell Receptor beta OR TS=T-Cell Receptor, alpha-beta OR TS=T-Cell Antigen receptor specificity OR TS=T-Cell Receptor specificity OR TS=T-Cell receptor repertoire | **153.693** |
| **#3 Combined search:** #1 AND #2 | **411** |

Database search was concluded in October 15, 2020 at 20:00 p.m.
