# Supplementary material for: T-cell receptor variable region usage in Chagas disease: A systematic review of experimental and human studies
Source: PLoS Negl Trop Dis. 2022 Sep 15;16(9):e0010546. doi: 10.1371/journal.pntd.0010546 (PMC9477334; doi:10.1371/journal.pntd.0010546)
Supplement: S2 Table — (DOCX) [file pntd.0010546.s002.docx]

**S2 Table. Assessment of the reporting quality of the preclinical studies.**

| **Studies**  **Quality criteria** | **Leite de Moraes et al., 1994 [1]** | **Cordeiro Silva et al., 1996 [2]** | **Cardoni et al., 1996 [3]** | **Sunnemark et al., 1998 [4]** | **Mendes-da-Cruz et al., 2003 [5]** | **Tekiel et al., 2005 [6]** | **Vogt et al., 2008 [7]** | **Criteria completed (n)** | **Criteria completed (%)** |
| --- | --- | --- | --- | --- | --- | --- | --- | --- | --- |
| **Title** |  |  |  |  |  |  |  |  |  |
| Accurate and concise description of the content of the article | √ | √ | √ | √ | √ | √ | √ | 7 | 100 |
| **Abstract** |  |  |  |  |  |  |  |  |  |
| Summary of the background, objectives, methods, main findings and conclusions | √ | √ | √ | √ | √ | √ | √ | 7 | 100 |
| **Background** |  |  |  |  |  |  |  |  |  |
| Sufficient scientific background | √ | No | √ | No | √ | √ | √ | 5 | 71 |
| Rational explanation of the experimental approach | √ | √ | √ | √ | √ | √ | √ | 7 | 100 |
| **Objectives** |  |  |  |  |  |  |  |  |  |
| Cleary primary and secondary objectives | No | No | √ | √ | √ | √ | √ | 5 | 71 |
| **Materials and Methods** |  |  |  |  |  |  |  |  |  |
| **Ethical statement** |  |  |  |  |  |  |  |  |  |
| Ethical review permissions, relevant licenses and official guidelines for use of animals | No | No | No | No | √ | No | √ | 2 | 28 |
| **Study design** |  |  |  |  |  |  |  |  |  |
| Numbers of animals per group | √ | √ | √ | √ | √ | √ | √ | 7 | 100 |
| Information on whether the experiment was performed as a blind controlled study | No | No | No | No | No | No | No | 0 | 0 |
| **Experimental procedures** |  |  |  |  |  |  |  |  |  |
| Parasite species | √ | √ | √ | √ | √ | √ | √ | 7 | 100 |
| Parasite strain | √ | √ | √ | √ | √ | √ | √ | 7 | 100 |
| Parasite inoculum | √ | √ | √ | √ | √ | √ | √ | 7 | 100 |
| Inoculum route | √ | √ | √ | √ | √ | √ | √ | 7 | 100 |
| Time of infection | √ | √ | √ | √ | √ | √ | √ | 7 | 100 |
| Rationale for choice of parasite inoculum | No | No | No | √ | No | √ | No | 2 | 28 |
| Rationale for choice of route of administration | No | No | No | No | No | √ | No | 1 | 14 |
|  |  |  |  |  |  |  |  |  |  |
| **Experimental animals** |  |  |  |  |  |  |  |  |  |
| Information regarding animals’ species | √ | √ | √ | √ | √ | √ | √ | 7 | 100 |
| Strain of the animal | √ | √ | √ | √ | √ | √ | √ | 7 | 100 |
| Sex of the animal | No | No | √ | √ | √ | √ | √ | 5 | 71 |
| Weight range of the animals | No | No | No | No | No | No | No | 0 | 0 |
| Age of the animals | √ | √ | √ | √ | √ | √ | √ | 7 | 100 |
| Information related to previous procedures performed on the animals | No | No | No | No | No | No | No | 0 | 0 |
| **Housing and husbandry** |  |  |  |  |  |  |  |  |  |
| Housing of experimental animals (facility, animals/cage, material) | No | No | No | No | No | No | No | 0 | 0 |
| Breeding program, light/dark cycle, temperature, quality of water | No | No | No | No | No | No | No | 0 | 0 |
| Welfare-related assessments before, during, or after the experiment | No | No | No | No | No | No | No | 0 | 0 |
| **Sample size** |  |  |  |  |  |  |  |  |  |
| Number of animals used for each experimental and group | √ | √ | √ | √ | √ | √ | √ | 7 | 100 |
| Explanation regarding number of animals and sample size calculation | No | No | No | No | No | No | No | 0 | 0 |
| **Allocating animals to experimental groups** |  |  |  |  |  |  |  |  |  |
| Full details of how animals were allocated to groups (randomization) | No | No | No | No | No | No | No | 0 | 0 |
| Order of animals inoculated and evaluation | No | No | No | No | No | No | No | 0 | 0 |
| **Experimental outcomes** |  |  |  |  |  |  |  |  |  |
| Clear experimental outcomes assessed | √ | √ | √ | √ | √ | √ | √ | 7 | 100 |
| **Statistical methods** |  |  |  |  |  |  |  |  |  |
| Statistical methods used for each analysis | No | No | No | No | √ | No | √ | 2 | 28 |
| Specification of the unit of analysis for each dataset | No | No | No | No | √ | No | √ | 2 | 28 |
| Methods used to assess adequacy of the statistical approach | No | No | No | No | √ | No | √ | 2 | 28 |
| **Results** |  |  |  |  |  |  |  |  |  |
| **Baseline data** |  |  |  |  |  |  |  |  |  |
| Description of health status of animals before inoculation | No | No | No | No | No | No | No | 0 | 0 |
| **Numbers analyzed** |  |  |  |  |  |  |  |  |  |
| Number of animals in each group included in each analysis | No | No | No | No | √ | √ | √ | 3 | 42 |
| Data not included in the analysis (explanation of exclusion) | No | No | No | No | No | No | No | 0 | 0 |
| **Outcomes and estimation** |  |  |  |  |  |  |  |  |  |
| Description of results (quality of text, tables, figures) | √ | √ | √ | √ | √ | √ | √ | 7 | 100 |
| Information statistical (Mean Standard± Deviation) | √ | √ | √ | No | √ | √ | √ | 6 | 85 |
| **Adverse events** |  |  |  |  |  |  |  |  |  |
| Information regarding mortality | No | √ | √ | No | No | No | No | 2 | 28 |
| Modifications to the protocols to reduce adverse events | No | No | No | No | No | No | No | 0 | 0 |
|  | | | | | |  |  |  |  |
| **Discussion** |  |  |  |  |  |  |  |  |  |
| **Interpretation/scientific implications** |  |  |  |  |  |  |  |  |  |
| Interpretation of the results, consider objectives, hypotheses, current theory | √ | √ | √ | √ | √ | √ | √ | 7 | 100 |
| Comments on limitations (bias, limitations of the model, imprecision of results) | No | No | No | No | No | No | No | 0 | 0 |
| **Generalizability/translation** |  |  |  |  |  |  |  |  |  |
| Comments on how the findings are likely to translate to other species and relevance to humans | No | No | No | No | No | No | No | 0 | 0 |
| **Funding** |  |  |  |  |  |  |  |  |  |
| List of funding sources and the role of the founder (s) in study | √ | √ | √ | √ | √ | √ | √ | 7 | 100 |
| **Criteria completed (n)** | **19** | **19** | **22** | **20** | **26** | **24** | **26** |  |  |
| **Criteria completed (%)** | **44,2** | **44,2** | **51** | **46** | **60** | **55** | **60** |  |  |

√: Criteria completed, No: criteria not completed.

**References:**

1. Leite-de-Moraes MDC, Coutinho A, Hontebeyrle-joskowicz M, Minoprio P, Eisen H, Bandeira A. Skewed Vβ TCR repertoire of CD8+ T cells in murine *Trypanosoma cruzi* infection. Int Immunol. 1994;6(3):387–92. doi: 10.1093/intimm/6.3.387.

2. Cordeiro da Silva A, Lima ECS, Vicentelli M-H, Minoprio P. Vβ6-bearing T cells are involved in resistance to *Trypanosoma cruzi* infection in XID mice. Int Immunol. 1996;8(8):1213–9. doi: 10.1002/acr.20380.

3. Cardoni RL, Antunez MI, Orn A, Grönvik KO. T cell receptor Vβ repertoire in the thymus and spleen of mice infected with *Trypanosoma cruzi*. Cell Immunol. 1996;169(2):238–45. doi: 10.1006/cimm.1996.0114.

4. Sunnemark D, Andersson R, Harris RA, Jeddi-Tehrani M, Örn A. Enhanced prevalence of T cells expressing TCRBV8S2 and TCRBV8S3 in hearts of chronically *Trypanosoma cruzi*-infected mice. Immunol Lett. 1998;60(2–3):171–7. doi: 10.1016/s0165-2478(97)00153-3.

5. Mendes-da-Cruz DA, De Meis J, Cotta-de-Almeida V, Savino W. Experimental *Trypanosoma cruzi* infection alters the shaping of the central and peripheral T-cell repertoire. Microbes Infect. 2003;5(10):825–32. doi: 10.1016/s1286-4579(03)00156-4.

6. Tekiel V, Oliveira GC, Correa-Oliveira R, Sánchez D, González-Cappa SM. Chagas’ disease: TCRBV9 over-representation and sequence oligoclonality in the fine specificity of T lymphocytes in target tissues of damage. Acta Trop. 2005;94(1):15–24. doi: 10.1016/s1286-4579(03)00156-4.

7. Vogt J, Alba Soto CD, Mincz MP, Mirkin GA. Impaired *Trypanosoma cruzi*-specific IFN-γ secretion by T cells bearing the BV9 T-cell receptor is associated with local IL-10 production in non-lymphoid tissues of chronically infected mice. Microbes Infect. 2008;10(7):781–90. doi: 10.1016/j.micinf.2008.04.01
