# Supplementary material for: T-cell receptor variable region usage in Chagas disease: A systematic review of experimental and human studies
Source: PLoS Negl Trop Dis. 2022 Sep 15;16(9):e0010546. doi: 10.1371/journal.pntd.0010546 (PMC9477334; doi:10.1371/journal.pntd.0010546)
Supplement: S3 Table — (DOCX) [file pntd.0010546.s003.docx]

**S3 Table. Evaluation of bias risk in studies with animal models according to the SYRCLE’s tool.**

| **Studies**  **Signaling questions** | | **Leite de Moraes et al., 1994 [1]** | **Cordeiro Silva et al., 1996 [2]** | **Cardoni et al., 1996 [3]** | **Sunnemark et al., 1998 [4]** | **Mendes-da-Cruz et al., 2003 [5]** | **Tekiel et al., 2005 [6]** | **Vogt et al., 2008 [7]** |
| --- | --- | --- | --- | --- | --- | --- | --- | --- |
| **1** | Was the allocation sequence adequately generated and applied? | No | No | No | No | No | No | No |
| **2** | Were the groups similar at baseline? | ? | ? | ? | ? | ? | ? | ? |
| **3** | Was the allocation to the different groups adequately concealed during? | No | No | No | No | No | No | No |
| **4** | Were the animals randomly housed during the experiment? | No | No | No | No | No | No | No |
| **5** | Were the caregivers and/or investigators blinded from knowledge which intervention each animal received during the experiment? | No | No | No | No | No | No | No |
| **6** | Were animals selected at random for outcome assessment? | ? | ? | ? | ? | ? | ? | ? |
| **7** | Was the outcome assessor blinded? | ? | ? | ? | ? | ? | ? | ? |
| **8** | Were incomplete outcome data adequately addressed? | Yes | Yes | Yes | Yes | Yes | Yes | Yes |
| **9** | Are reports of the study free of selective outcome reporting? | Yes | Yes | Yes | Yes | Yes | Yes | Yes |
| **10** | Was the study apparently free of other problems that could result in high risk of bias? | ? | ? | ? | ? | Yes | Yes | Yes |

Yes: low risk of bias; No: high risk of bias; ?: unclear risk of bias. It is not recommend calculating a summary score for each individual study when using this toll.

**S3.1 Table. Analysis of methodological bias in animal studies.**

| **Study** | **1** | **2** | **3** | **4** | **5** | **6** | **7** | **8** | **9** | **10** |
| --- | --- | --- | --- | --- | --- | --- | --- | --- | --- | --- |
|  | **Selection bias 1** | **Selection bias 2** | **Selection bias 3** | **Performance bias 1** | **Performance bias 2** | **Detection bias 1** | **Detection bias 2** | **Attrition bias** | **Reporting bias** | **Other potential bias** |
| **Leite de Moraes et al. 1994 [1]** | x | ? | x | x | x | ? | ? | ✓ | ✓ | ? |
| **Cordeiro Silva et al 1996 [2]** | x | ? | x | x | x | ? | ? | ✓ | ✓ | ? |
| **Cardoni et al. 1996 [3]** | x | ? | x | x | x | ? | ? | ✓ | ✓ | ? |
| **Sunnemark et al. 1998 [4]** | x | ? | x | x | x | ? | ? | ✓ | ✓ | ? |
| **Mendes-da-Cruz et al. 2003[5]** | x | ? | x | x | x | ? | ? | ✓ | ✓ | ✓ |
| **Tekiel et al. 2005 [6]** | x | ? | x | x | x | ? | ? | ✓ | ✓ | ✓ |
| **Vogt et al. 2008 [7]** | x | ? | x | x | x | ? | ? | ✓ | ✓ | ✓ |

1 ✓= Adequate randomization; ?= randomized but no details; x=no evidence of randomization. 2 ✓= Baseline characteristics given; ?= insufficient baseline characteristics; x= baseline characteristics not given. 3 ✓= Evidence of adequate concealment of groups; ?= unknown of adequate concealment of groups; x= no evidence of adequate concealment of groups. 4 ✓= Evidence of random housing of animals; ?= unknown housing arrangement. 5 ✓= Evidence of caregivers blinded to intervention; x= no evidence of caregivers blinded to intervention. 6 ✓= Evidence of random selection for assessment; ?= Evidence of random selection for assessment; x= no evidence of random selection for assessment. 7 ✓= Evidence of assessor blinded; ?= unknown of assessor blinded x= no evidence of assessor blinded. 8 ✓= Explanation of missing animal data; ?= unknown of missing animal data; x=no explanation of missing animal data. 9 ✓= Free of selective reporting based on methods/results; ?= inconclusive reporting; x= selective reporting. 10 ✓=Free of other high bias risk; ?= insufficient data to determine risk of other bias.

**References:**

1. Leite-de-Moraes MDC, Coutinho A, Hontebeyrle-joskowicz M, Minoprio P, Eisen H, Bandeira A. Skewed Vβ TCR repertoire of CD8+ T cells in murine *Trypanosoma cruzi* infection. Int Immunol. 1994;6(3):387–92. doi: 10.1093/intimm/6.3.387.

2. Cordeiro da Silva A, Lima ECS, Vicentelli M-H, Minoprio P. Vβ6-bearing T cells are involved in resistance to *Trypanosoma cruzi* infection in XID mice. Int Immunol. 1996;8(8):1213–9. doi: 10.1002/acr.20380.

3. Cardoni RL, Antunez MI, Orn A, Grönvik KO. T cell receptor Vβ repertoire in the thymus and spleen of mice infected with *Trypanosoma cruzi*. Cell Immunol. 1996;169(2):238–45. doi: 10.1006/cimm.1996.0114.

4. Sunnemark D, Andersson R, Harris RA, Jeddi-Tehrani M, Örn A. Enhanced prevalence of T cells expressing TCRBV8S2 and TCRBV8S3 in hearts of chronically *Trypanosoma cruzi*-infected mice. Immunol Lett. 1998;60(2–3):171–7. doi: 10.1016/s0165-2478(97)00153-3.

5. Mendes-da-Cruz DA, De Meis J, Cotta-de-Almeida V, Savino W. Experimental *Trypanosoma cruzi* infection alters the shaping of the central and peripheral T-cell repertoire. Microbes Infect. 2003;5(10):825–32. doi: 10.1016/s1286-4579(03)00156-4.

6. Tekiel V, Oliveira GC, Correa-Oliveira R, Sánchez D, González-Cappa SM. Chagas’ disease: TCRBV9 over-representation and sequence oligoclonality in the fine specificity of T lymphocytes in target tissues of damage. Acta Trop. 2005;94(1):15–24. doi: 10.1016/s1286-4579(03)00156-4.

7. Vogt J, Alba Soto CD, Mincz MP, Mirkin GA. Impaired *Trypanosoma cruzi*-specific IFN-γ secretion by T cells bearing the BV9 T-cell receptor is associated with local IL-10 production in non-lymphoid tissues of chronically infected mice. Microbes Infect. 2008;10(7):781–90. doi: 10.1016/j.micinf.2008.04.01
