# Supplementary material for: T-cell receptor variable region usage in Chagas disease: A systematic review of experimental and human studies
Source: PLoS Negl Trop Dis. 2022 Sep 15;16(9):e0010546. doi: 10.1371/journal.pntd.0010546 (PMC9477334; doi:10.1371/journal.pntd.0010546)
Supplement: S4 Table — (DOCX) [file pntd.0010546.s004.docx]

**S4 Table. Bias analysis of human studies of according to the Downs and Black Quality Index^1^.**

| **Studies**  **Signaling questions** | **Cunha-Neto et al., 1994 [1]** | **Costa et al., 2000 [2]** | **Fernández et al., 2002 [3]** | **Hermann et al., 2002 [4]** | **Menezes et al., 2004 [5]** | **Menezes et al., 2012 [6]** |
| --- | --- | --- | --- | --- | --- | --- |
| Is the hypothesis/aim/objective of the study clearly described? | 1 | 1 | 1 | 1 | 1 | 1 |
| Main outcomes were clearly described in the Introduction or Methods? | 1 | 0 | 0 | 1 | 1 | 1 |
| Are the characteristics of the patients included in the study clearly described? | 1 | 1 | 1 | 1 | 1 | 1 |
| Interventions were clearly described? | 1 | 1 | 1 | 1 | 1 | 1 |
| Distributions of confounders in each group compared was clearly described? | 1 | 1 | 1 | 1 | 1 | 1 |
| Are the main findings of the study clearly described? | 1 | 1 | 1 | 1 | 1 | 1 |
| Provide estimates of the random variability in the data for the main outcomes? | 1 | 1 | 1 | 1 | 1 | 1 |
| The important adverse events that may be a consequence of the intervention been reported? | 0 | 0 | 0 | 0 | 0 | 0 |
| The characteristics of patients lost to follow-up been described? | 0 | 0 | 0 | 0 | 0 | 0 |
| Have actual probability values been reported for the main outcomes? | 0 | 1 | 1 | 1 | 1 | 1 |
| The subjects were representative of the entire population from which they were recruited? | 1 | 1 | 1 | 1 | 1 | 1 |
| Were the subjects prepared to participate representative of the entire population from which they were recruited? | 0 | 1 | 1 | 1 | 1 | 1 |
| Were the staff, places, and facilities, representative of the treatment the majority of patients receive? | 0 | 1 | 1 | 1 | 1 | 1 |
| Was an attempt made to blind study subjects to the intervention? | 0 | 0 | 0 | 0 | 0 | 0 |

| Was an attempt made to blind those measuring the main outcomes of the intervention? | 0 | 0 | 0 | 0 | 0 | 0 |
| --- | --- | --- | --- | --- | --- | --- |
| Results based on “data dredging”, were clearly reported? | 0 | 1 | 1 | 1 | 1 | 1 |
| Do the analysis adjust for different lengths (follow-up or period between the intervention and outcome)? | 1 | 1 | 1 | 1 | 1 | 1 |
| Were the statistical tests used to assess the main outcomes appropriate? | 1 | 1 | 1 | 1 | 1 | 1 |
| Was compliance with intervention/s reliable? | 1 | 1 | 1 | 1 | 1 | 1 |
| Were the main outcome measures used accurate (valid and reliable)? | 1 | 1 | 1 | 1 | 1 | 1 |
| Were the patients in different groups or were the cases and controls recruited from the same population? | 1 | 1 | 1 | 1 | 1 | 1 |
| Were study subjects in different groups or were the cases and controls recruited over the same period of time? | 0 | 0 | 0 | 0 | 0 | 0 |
| Were study subjects randomized in groups? | 0 | 0 | 1 | 0 | 0 | 0 |
|  |  |  |  |  |  |  |
| Randomized intervention assignment concealed from patients and health care staff until recruitment was complete? | 0 | 0 | 0 | 0 | 0 | 0 |
| Adequate adjustment for confounding from which the main findings were drawn? | 0 | 1 | 1 | 1 | 1 | 1 |
| Losses of patients were considered? | 0 | 0 | 0 | 0 | 0 | 0 |
| **Quality Score/items (n)** | **13** | **17** | **18** | **18** | **18** | **18** |
| **Quality Score/items (%)** | **50** | **65** | **69** | **69** | **69** | **69** |
|  |  |  |  |  |  |  |

References:

1. Cunha-Neto E, Moliterno R, Coelho V, Guilherme L, Bocchi E, Higuchi MDL, et al. Restricted heterogeneity of T cell receptor variable alpha chain transcripts in hearts of Chagas’disease cardiomyopathy patients. Parasite Immunol. 1994;16(4):171–9. doi: 10.1111/j.1365-3024.1994.tb00337.x.

2. Costa RP, Gollob KJ, Fonseca LL, Rocha MOC, Chaves ACL, Medrano-Mercado N, et al. T-Cell Repertoire Analysis in Acute and Chronic Human Chagas’Disease: Differentail Frequencies of Vb5 Expressing T Cells. Scand J Immunol. 2000;51(5):511–9. doi:10.1046/j.1365-3083.2000.00706.x

3. Fernández-Mestre MT, Jaraquemada D, Bruno RE, Caro J, Layrisse Z. Analysis of the T-cell receptor β-chain variable-region (Vβ) repertoire in chronic human Chagas’ disease. Tissue Antigens. 2002;60(1):10–5. doi:10.1034/j.1399-0039.2002.600102.x

4. Hermann E, Truyens C, Alonso-Vega C, Even J, Rodriguez P, Berthe A, et al. Human fetuses are able to mount an adultlike CD8 T-cell response. Blood. 2002;100(6):2153–8.

5. Menezes CAS, Rocha MOC, Souza PEA, Chaves ACL, Gollob KJ, Dutra WO. Phenotypic and functional characteristics of CD28+ and CD28− cells from chagasic patients: distinct repertoire and cytokine expression. Clin Exp Immunol. 2004;137(1):129–38. doi: 10.1111/j.1365-2249.2004.02479.x

6. Menezes CAS, Sullivan AK, Falta MT, Mack DG, Freed BM, C Rocha MO, et al. Highly conserved CDR3 region in circulating CD4 + Vb5 + T cells may be associated with cytotoxic activity in Chagas disease. Clin Exp Immunol. 2012;109–18. doi: 10.1111/j.1365-2249.2012.04608.x
